# Spatial Assortment of Mixed Propagules Explains the Acceleration of Range Expansion

**DOI:** 10.1371/journal.pone.0103409

**Published:** 2014-08-08

**Authors:** Andriamihaja Ramanantoanina, Aziz Ouhinou, Cang Hui

**Affiliations:** 1 Centre for Invasion Biology, Department of Mathematical Sciences, Stellenbosch University, Matieland, South Africa; 2 Mathematical and Physical Biosciences, African Institute for Mathematical Sciences, Muizenberg, South Africa; 3 Department of Mathematics, Faculty of Sciences and Technology, University of Sultan Moulay Slimane, Beni-Mellal, Morocco; CNRS/Université Joseph-Fourier, France

## Abstract

Range expansion of spreading organisms has been found to follow three types: (i) linear expansion with a constant rate of spread; (ii) bi-phase expansion with a faster linear expansion following a slower linear expansion; and (iii) accelerating expansion with a continuously increasing rate of spread. To date, no overarching formula exists that can be applied to all three types of range expansion. We investigated how propagule pressure, i.e., the initial number of individuals and their composition in terms of dispersal ability, affects the spread of a population. A system of integrodifference equations was then used to model the spatiotemporal dynamics of the population. We studied the dynamics of dispersal ability as well as the instantaneous and asymptotic rate of spread. We found that individuals with different dispersal abilities were spatially sorted with the stronger dispersers situated at the expanding range front, causing the velocity of expansion to accelerate. The instantaneous rate of spread was found to be fully determined by the growth and dispersal abilities of the population at the advancing edge of the invasion. We derived a formula for the asymptotic rate of spread under different scenarios of propagule pressure. The results suggest that data collected from the core of the invasion may underestimate the spreading rate of the population. Aside from better managing of invasive species, the derived formula could conceivably also be applied to conservation management of relocated, endangered or extra-limital species.

## Introduction

The ability to estimate the rate of spread of an invasive species is important for the success of its management and control [1]. Early theories suggested that the velocity at which a species expands its range depends on the population growth and dispersal rates [2]. Models based on partial differential equations, specifically the reaction-diffusion (RD) model, assume a normal distribution of species' dispersal distances (i.e. dispersal kernel) and yield a widely-used formula which depicts a constant rate of spread (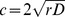, where 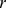 and 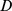 denote the intrinsic growth and diffusion rates, respectively) [2]–[4]. However, a growing body of evidence suggests that the rate of spread for most species may not be constant [5]. Shigesada et al. [6] group patterns of range expansion into three types: type I, linear expansion with a constant rate of spread; type II, bi-phase expansion with a faster linear expansion following a slower linear expansion; type III, accelerating expansion with a continuously increasing rate of spread.

To fully comprehend the accelerating nature of type II and III range expansion, different dispersal strategies have been incorporated into RD models. In particular, fat-tailed dispersal kernels (i.e. movements with a substantial portion of long-distance dispersal) have been shown to be capable of boosting the range expansion and are, thus, an appropriate mechanism for explaining the accelerating range expansion [7]. However, this explanation suffers from two pitfalls [8]. First, the rate of spread predicted from a fat-tailed dispersal kernel will keep increasing without an upper bound, an obvious exaggeration of the reality. Second, estimating the parameters of a fat-tailed dispersal kernel is difficult due to the obvious rarity of long-distance dispersal events [9] and often requires a substantial amount of recapturing records [10]. Clark et al. [11] tackle the conundrum of type II biphasic and type III accelerating range expansion by using a combined dispersal kernel, with the individual having a probability of 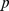 to move a short distance and a probability of 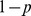 to move a long distance (see also [6], [12]). This combined dispersal kernel can lead to a budding pattern of stratified range expansion, with the expansion speeding up when the buds of founding populations merge into a super colony. Such stratified dispersal is especially common in species with multiple stages (e.g. species with dispersal polymorphism or different dispersal strategies at different instars; [13]) or with multiple dispersal vectors (e.g. combined wind and animal-born seed dispersal; [14]). Introduced species often experience stratified dispersal due to the additional human-facilitated translocation [15]–[19].

Two recent insights from invasion biology sketch a new alternative concept that could explain the range expansion that accelerates to a limited speed. First, propagule size (i.e., the number of individuals released into an introduced area) has been identified as one key factor of invasion success [20]–[23]. A large propagule size can efficiently counteract the positive density dependence caused by the Allee and founder effects that hamper the establishment of initial propagules in a novel ecosystem [24]–[26]. More importantly, studies show that the initial propagule often consists of a suite of individuals with different performance ability [25], [27]–[29], and assuming propagules with identical traits often leads to an underestimation of the spreading rate in animals [30]. It is, thus, more reasonable to conceptualize the initial propagule as a group of individuals with differences in their life-history traits.

Second, dispersal strategy is a density- and context-dependend adaptive trait. Overwhelming cases support a positive density-dependent dispersal [31], and we expect to see a higher dispersal rate at the core high-density population than at the low-density marginal population. The interplay of local adaptation and environmental gradient can further lead to a context-dependent dispersal [32]–[34], with dispersal strategy and spreading behaviour highly sensitive to the spatiotemporal variability of habitat quality, especially during range shifts [35]–[38]. Besides the commonness of the density- and context-dependent dispersal strategy, in many invasive species, the advancing range front poses an additional selection force of spatial gradient, only existing temporarily while the range expansion is ongoing. Individuals will be sorted along this non-equilibrium spatial gradient according to their dispersal abilities, with individuals having stronger dispersal abilities more likely to locate at the advancing range front [39], [40]. Examples of spatial sorting at the advancing range front are accumulating in literature, such as developing longer legs in cane toads (*Bufo marinus*) in Australia [41]; changing wing shape of Indian mynas (*Acridotheres tristis*) in South Africa [42], [43], and more long-winged morphs of bush crickets in UK [44]. The range expansion could be accelerating due to a dynamic dispersal kernel driven by this process of spatial sorting for stronger dispersers at the range front. Indeed, based on recapturing records and metrics of spatial genetics, Hui et al. [10] and Berthouly-Salazar et al. [45] further demonstrate a changing dispersal kernel through spatial sorting in the invasive European starling (*Sturnus vulgaris*) to support its accelerating range expansion in southern Africa.

Given these two insights from invasion biology, the RD model needs to be revisited and the formula 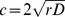 revised so that the velocity of accelerating range expansion can be accurately predicted. Here, we present a mathematical model that uses integrodifference equations to incorporate individuals with different dispersal abilities in the initial propagule. The new formulae of the instantaneous and asymptotic rates of spread derived from this model include not only rates of growth and dispersal as in the formula for linear expansion, but also parameters depicting the propagule size, its composition and the process of spatial sorting. The classic formula 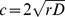 is shown to be a special case of the new ones. We further develop numerical simulations to test the performance of these formulae and advocate the use of them in the modelling and risk assessment of invasive species [46], as well as in forecasting the range shift of species in response to environmental changes [47], [48].

## The Model

For simplicity, we consider the invasion of a one-dimensional habitat by the mixed propagule which consists of *n* types of individuals with different dispersal abilities. Let *u_i_*(*x*,*t*) (*i* = 1,…, *n*) denote the population size of type *i* individuals at location *x* and time *t*. The dispersal of type *i* individuals is depicted by the dispersal kernel 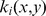 (i.e. the probability that a type *i* individual moves from location *y* to *x* during a time step [7]). Dispersal can be density dependent [49] and sensitive to habitat quality [10], complicating the formulation of the spreading rate. Consequently, we assume homogenous habitat and density-independent dispersal; that is, the dispersal kernel *k_i_* depends only on the distance between the locations (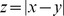). Let 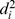 represent the variance of the dispersal kernel *k_i_*(*z*). For a Gaussian kernel, we have 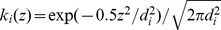, and for a Laplace kernel, 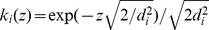. Hereafter 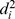 will be referred to as the dispersal ability of type *i* individuals.

The recruitment of type *i* individuals is governed by a non-negative function of population change rate 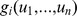. We assume that the dispersal ability is inheritable as in an asexual population [50], [51]; that is *g_i_*(*u*_1_,…, *u_n_*) = 0 if *u_i_* = 0. We further assume that the population does not suffer from the Allee effect and that the population change rate thus reflect the negative density dependence; that is, *g_i_*(*u*_1_,…, *u_n_*)≤*R_i_u_i_*, where *R_i_* (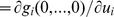) is the population growth rate. We also assume that there is no trade-off between the dispersal ability and the population growth rate; that is, individuals with different dispersal abilities have an equal population growth rate, *R*_1_ = … = *R_n_* = *R*. Examples of such population change functions include the Richer model [52], 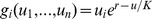, where 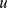 is the total population (Σ*u_i_*), and *r* ( = ln*R*) the intrinsic population growth rate.

Given the population size 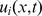 at time 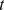, the population size at time 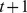 can be depicted by the following integrodifference equation [7],
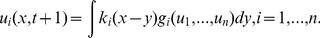
(1)The total population is governed by
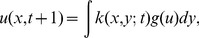
(2)where 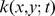 ( = 
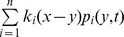
) is the *expected dispersal kernel* at location *y* and time *t*, and 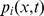 the proportion type *i* individuals in the mixed population at location *x* and time *t*, 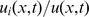. For the Ricker model, we have 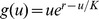. Evidently, the expected dispersal kernel not only depends on the dispersal distance (|*x*-*y*|) but also the population composition *p_i_*(*y*,*t*) where the individuals are dispersed from, suggesting a dynamic dispersal kernel [8].

Let the initial propagule consist of *U*_0_ individuals. We consider two specific scenarios of mixing individuals with different dispersal abilities in the initial propagules. The first scenario assumes that the initial propagule only consists of two types of individuals, with their dispersal abilities 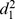 and 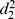 (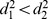). More specifically, we assume that the majority of the initial propagule are type 1 individuals with the dispersal ability 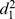, and only a small proportion of individuals have the dispersal ability 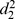; that is, 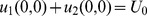, 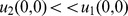 and 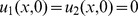 for 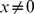.

The second scenario assumes individuals with many more dispersal abilities in the initial propagule. As the log-normal distribution has been confirmed for many species- and trait-level frequency distributions in ecological communities [53], [54], we generated the dispersal abilities of the initial propagules as follows. First, for each individual, a dispersal ability 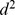 was randomly drawn from a log-normal distribution 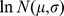 (hereafter, the standard deviation 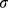 will be referred to as the *propagule diversity*). Note that the parameter 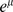 gives the median dispersal ability of the initial propagules. Second, we group the individuals into *n* types, with the type *i* individuals having the dispersal abilities between 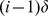 and 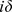, with 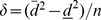, and 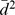 and 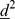 denote the maximal and minimal dispersal abilities, respectively. For simplicity, all individuals of type *i* were assigned the same dispersal ability 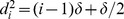.

The model was solved using the Fast Fourier Transform algorithm implemented in the SciPy library of Python [55]. To simplify the illustration, we call a type *i* individual a slower dispersal if 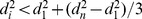, an intermediate disperser if 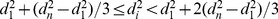, a fast disperser if 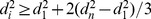. The shape of the expected dispersal kernel *k* and the *mean dispersal ability* of the individuals at location *x* and time *t*, 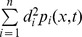 were investigated. The instantaneous and average rates of spread at time *t* were calculated as 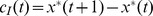 and 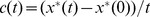, respectively, where 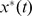 is the location of the range front defined for a certain threshold of detection 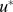 as 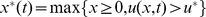. Recall that we are interested in the spread of the total population *u*(*x*,*t*).

## Results

### Propagule with two dispersal abilities

**Figure 1:**
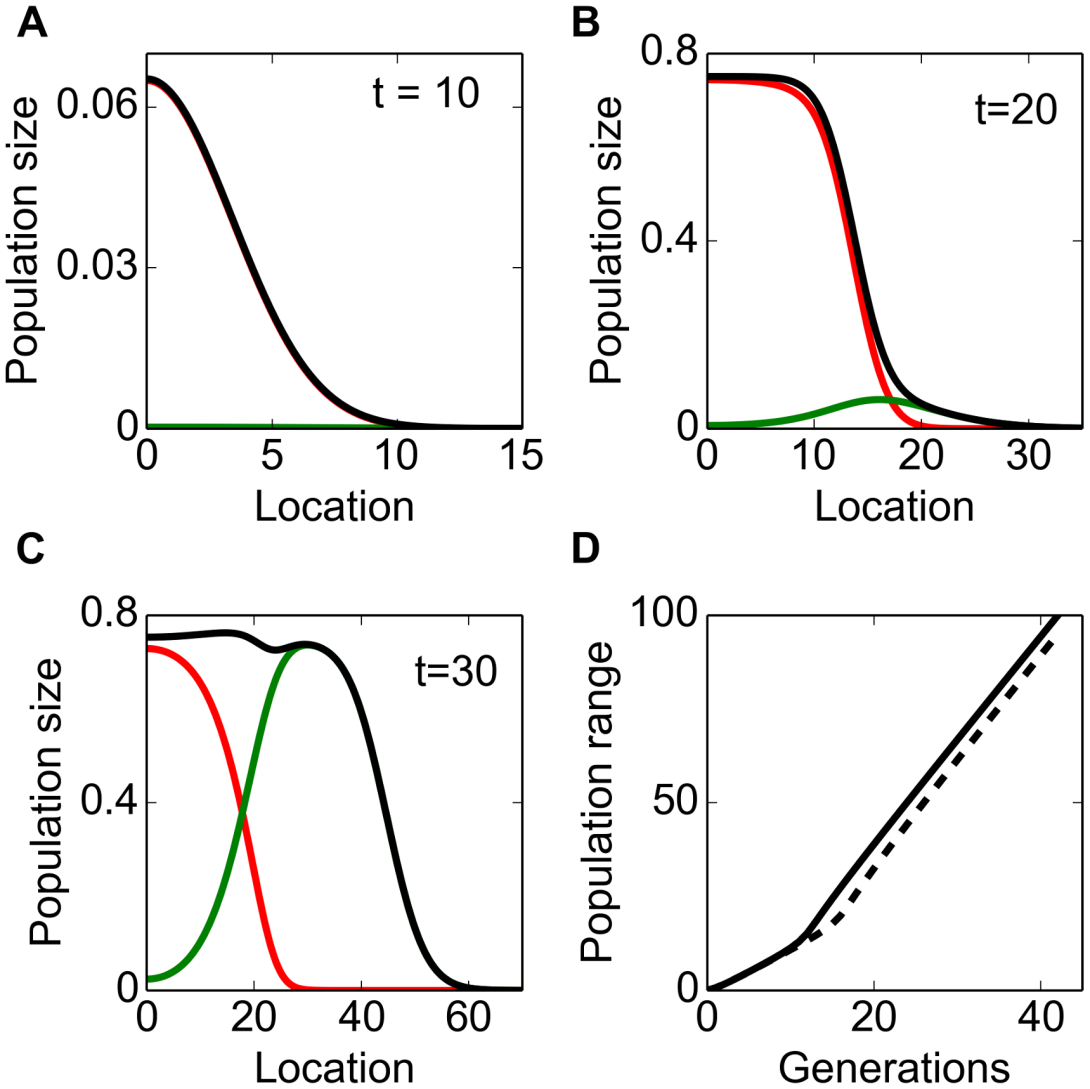
Spread of a population with two dispersal abilities. Parameter values are 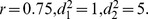 A–C: Population size at different time with the initial populations 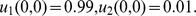 The colours red, green and black correspond to the type-1, type-2 and total population size respectively. D: A break of slope was observed in the population range. Initial propagules are 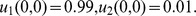 (solid line) and 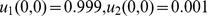 (dashed line).

When the initial propagule contains two types of individuals, the number of the fast disperser type 2 individuals remained low at the initial phase, while the total population consisted mainly of type 1 individuals, similar to the composition in the initial propagules (Fig. 1A). The type 2 individuals gradually reached the expanding front through spatial sorting (Fig. 1B) and then increased in numbers, with the type 1 individuals compressed back to the range core (comparing Fig. 1B with Fig. 1C). A breaking point of slope was observed during the range expansion (Fig. 1D), indicating a type II bi-phase expansion. The time lag to the breaking point decreased when more type 2 individuals were in the initial propagule but the asymptotic rate of spread was not affected by the propagule composition (Fig. 1D).

**Figure 2:**
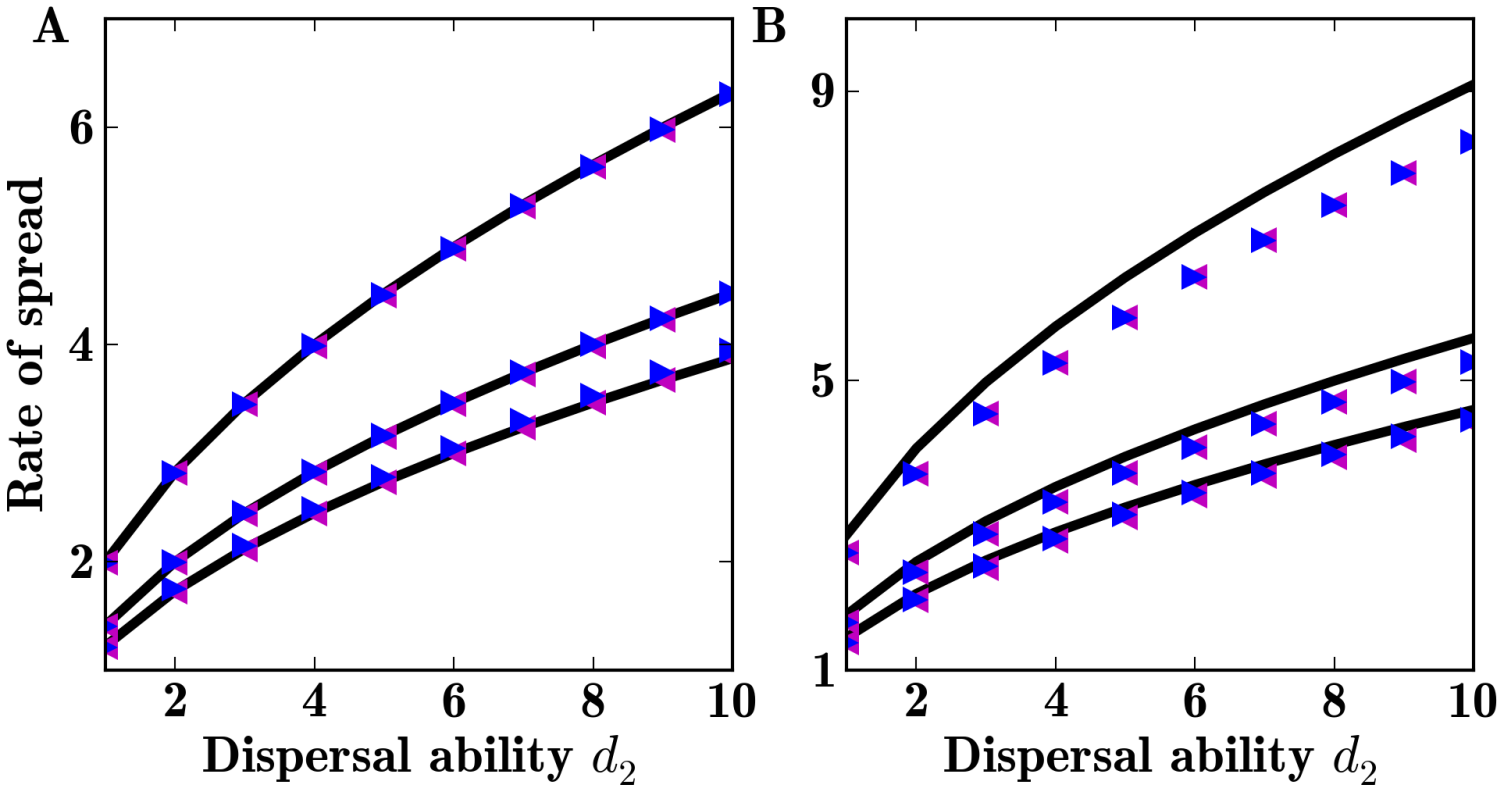
Asymptotic rate of spread of a population with two dispersal abilities. Theoretical (black lines) and computed asymptotic rate of spread when two dispersal abilities are present in the population. Initial propagules are 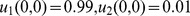 (blue triangle) and 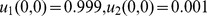 (magenta triangle). Other parameter values are 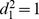 (1) 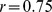 (2) 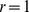 and (3) 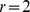. A: Using Gaussian dispersal kernels. B: Using Laplace dispersal kernels.

The asymptotic rate of spread for type II bi-phase expansion (i.e. the rate of spread at the second phase of expansion) was heuristically derived as follows. We note that the solution of the model (Eq.(1)) becomes a travelling wave of the 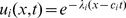 which spreads at the following rate:
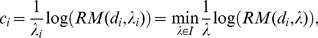
(3)where 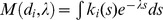 is the moment generating function of *k_i_*, and the interval *I* is 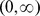 for Gaussian kernels and is 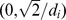 for Laplace kernels [7], [56]. Recall that we are interested in the spread of the total population 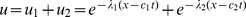. Because 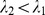 and 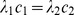 for Gaussian and Laplace kernels, we have 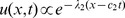 as *x* approaches infinity, meaning that the spreading rate of the total population (Eq.(2)) is given by 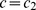. An approximation has been derived by Lutscher [57]:
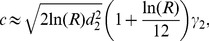
(4)where 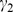 is the kurtosis of the dispersal kernel 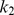. In particular, for Gaussian kernels we have:
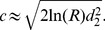
(5)For Laplace dispersal kernels we have:
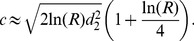
(6)The derived rate of spread *c* fits well with the asymptotic rate of spread obtained from the numerical simulations with a Gaussian dispersal kernel and also sets a close upper bound for the rate of spread with a Laplace dispersal kernel (Fig. 2).

### Propagules with multiple dispersal abilities

**Figure 3:**
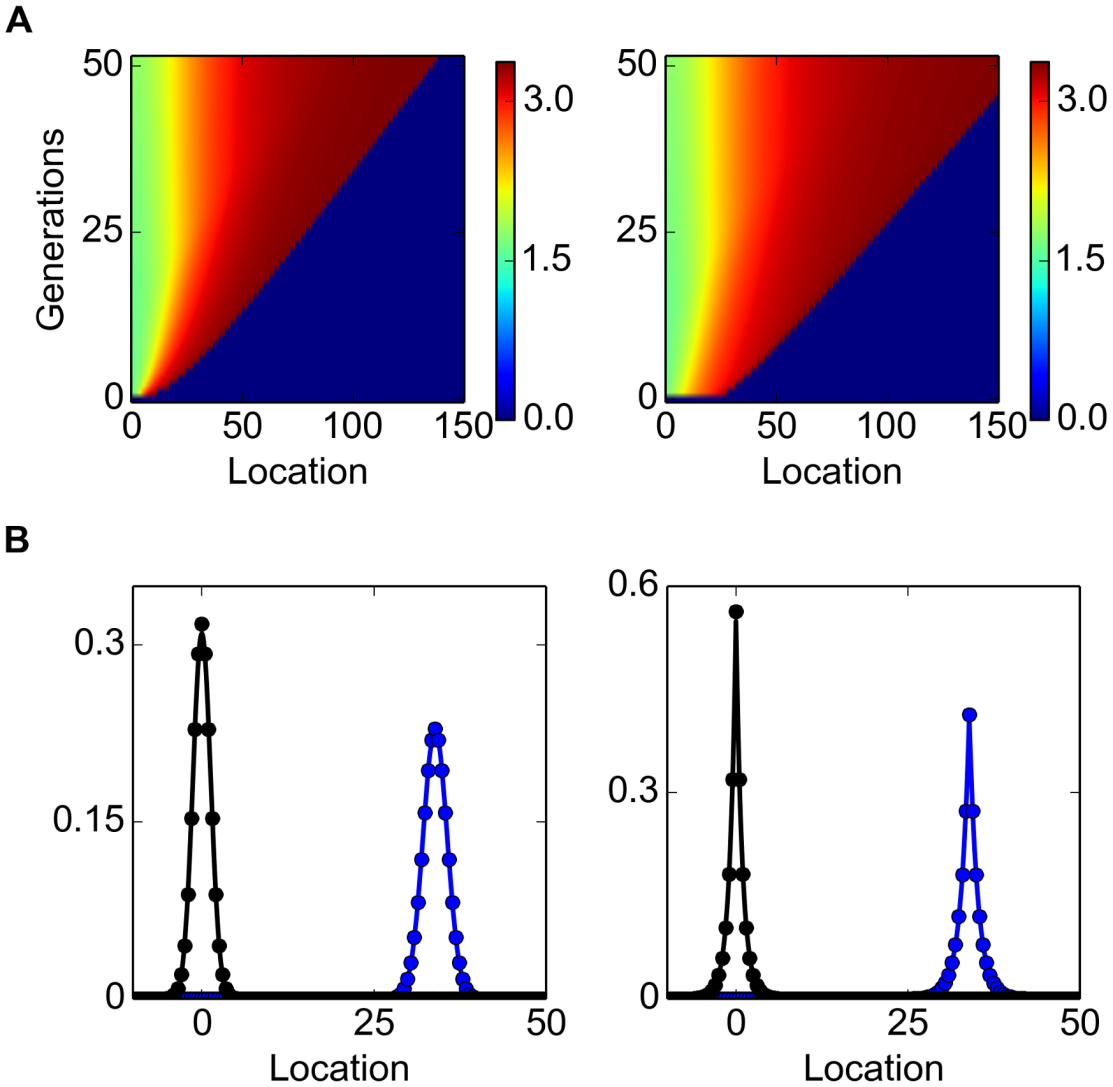
A propagating population with many dispersal abilities. The parameter values are 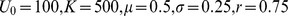. The colours red, green and blue correspond respectively to slow, intermediate and fast dispersers whereas the black lines represent the total population size at different time.

**Figure 4:**
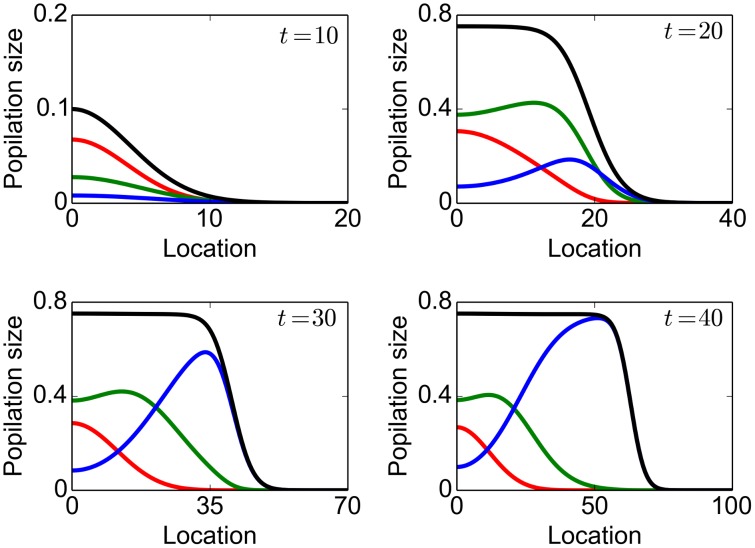
Evolution of the mean dispersal ability and expected dispersal kernels. (A–B) Mean dispersal ability: During the invasion, the dispersal ability at the front is higher than the dispersal ability at the core of the invasion. The region in blue indicates habitats that have not been invaded at each generation. (C–D): The expected dispersal kernel at the core of the invasion (in black) is narrower than that at the front of the invasion (blue). In both cases, the expected dispersal kernel (full circles) can be approximated by the kernel associated to the mean dispersal ability (Eq (8) and Eq (9)) (Solid lines). Figures (A,C) and (B,D) were obtained using Gaussian and Laplace dispersal kernels respectively.

When a number of *n* dispersal abilities were present in the initial propagule, the process of spatial sorting gradually pushed fast dispersers to the advancing range front while compressing slow dispersers to the range core (Fig. 3). Spatial sorting was also detected by calculating the mean dispersal ability which kept increasing while expanding (Fig. 4A–B). The expected dispersal kernel has a fatter tail at the range front than at the range core (Fig. 4C–D); that is, individuals at the front are more likely to travel longer distances than individuals from the range core. Importantly, the expected dispersal kernel was found closely related to the dispersal kernel corresponding to the mean dispersal ability. Consequently, for the expected Gaussian dispersal kernel can be approximated as,
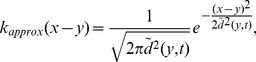
(8)and the expected Laplace dispersal kernel can be approximated as,
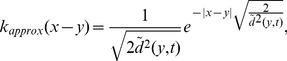
(9)where 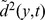 is the mean dispersal ability at location *y* and time *t*.

**Figure 5:**
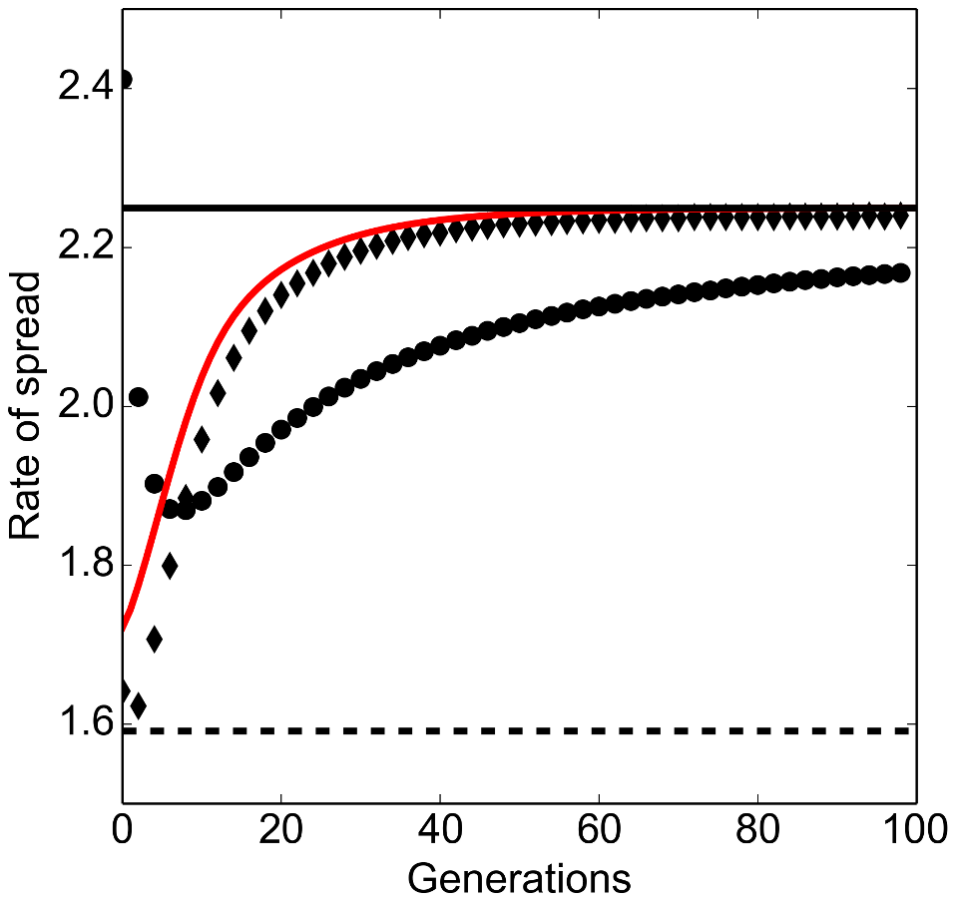
Rate of spread of a mixed population. Parameter values are 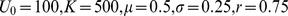. Diamonds: Instantaneous rate of spread. Circles: Average rate of spread. Solid black line: Predicted asymptotic rate of spread. Solid red line: predicted instantaneous rate of spread (Eq (10)). The rate of spread predicted by a single population model is shown by the dashed line.

We observed two distinct phases during the range expansion. The instantaneous rate of spread increased with time in the first phase (e.g. in the first 40 generations of the simulated population in Fig. 5), followed by a constant rate of spread in the second phase. Numerical simulations further suggested that the instantaneous rate of spread can be estimated by the mean dispersal ability of the population at the advancing range front (Fig. 5). Specifically, the instantaneous rate of spread *c* ^*^(*t*) can be approximated as the rate of spread of a single species with the kernel 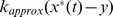:
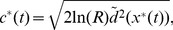
(10)where 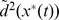 is the mean dispersal ability at the front 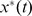 of the invasion at time *t*. Furthermore, following the same procedure as for the scenario with two dispersal abilities (section 3.1), we found that both the instantaneous and average rate of spread approached a same asymptotic rate of spread:
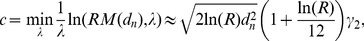
(11)where 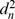 is the maximal dispersal ability in the population.

**Figure 6:**
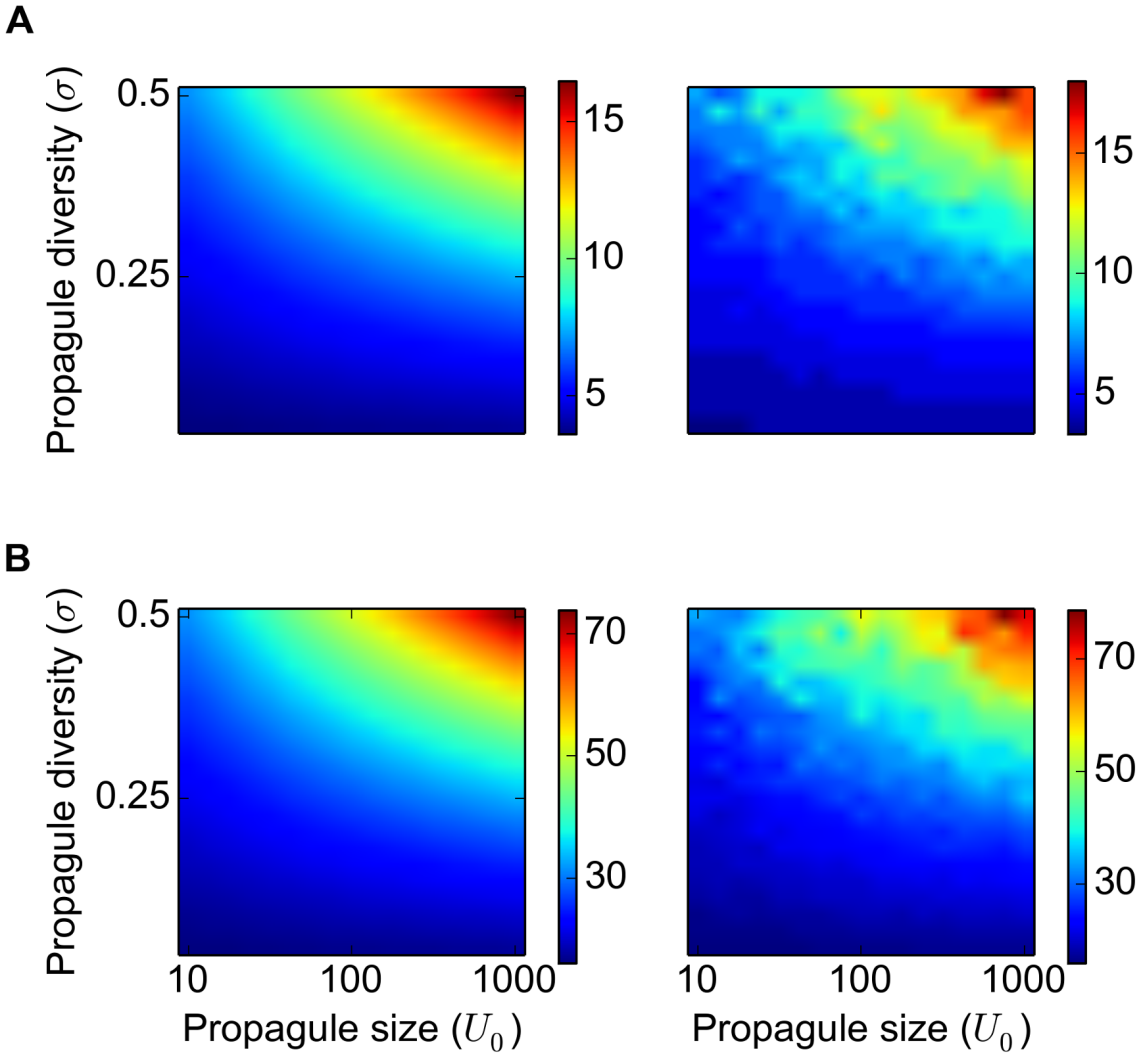
Propagule pressure and the median rate of spread. Theoretical (left) and computed (right) median of the asymptotic rate of spread for 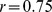, A: 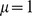 and B: 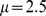. The computed median was obtained from 15 simulations for each set of parameter values.

As the dispersal abilities of the individuals were randomly drawn from the lognormal distribution, 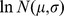, the maximal dispersal ability 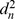 is a random number. Let 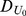 be the random variable of the maximal dispersal ability in the initial propagules of size 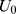. The cumulative distribution function of 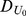 can be given as, 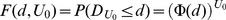, where 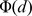 is the cumulative distribution function of the lognormal distribution, 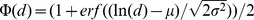, where *erf*(·) stands for the Gaussian error function. By solving 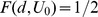 with respect to *d* we can obtain the median of 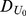:
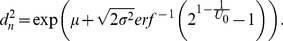
(12)For Gaussian dispersal kernels with propagules having multiple dispersal abilities, the asymptotic rate of spread is, thus, given by:
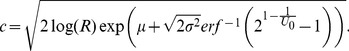
(13)This formula for the median asymptotic rate of spread was tested by solving Eq.(2) numerically for different propagule size *U*_0_ and propagule diversity *σ*. For each pair of *U*_0_ and *σ* the median of the asymptotic rate of spread from 15 simulations was calculated and compared with Eq.(13). Evidently, as the propagule diversity *σ* approaches zero (i.e. all individuals have an equal dispersal ability) the asymptotic rate of spread becomes independent of the propagule size, whereas increasing either propagule size or propagule diversity will result in a faster spreading rate (Fig. 6).

## Discussion

The role of propagule pressure in the introduction and establishment of introduced species has a rich background in literature [21]–[23]. In this work, we went further and investigated the contribution of propagule pressure in the actual rate of spread and the shape of the range expansion pattern. Two properties of the propagule were incorporated in the models, namely the propagule size (i.e. number of individuals in the propagule) and the composition of the propagule, namely the distribution of the dispersal abilities of the individuals in the initial propagule. Such propagule features were incorporated in our model using integrodifference equations (IDE).

We considered two compositional structures of the initial propagule. In the first case, we examined the importance of a small number of individuals with stronger dispersal ability in the propagule. A linear range expansion was observed during the initial phase of the invasion, which was followed by another linear expansion with a higher rate of spread. In other words, a sudden increase in the rate of spread can result from a mixed composition of the propagule. Furthermore, while the duration and rate of spread of the initial slow phase depended on the frequency of fast dispersers in the initial propagule, the long-term rate of spread of the population was determined by their dispersal ability, however rare they were in the initial propagule (Eq (7)). This phenomena is expected when the fast dispersers do not go extinct, for example as result of demographic stochasticity which are important in populations at low density [58], [59]. In the second case, we investigated the case where the dispersal abilities of the initial propagule are log-normally distributed. Dispersal abilities were subject to spatial sorting. More clearly, the expanding front was inhabited by individuals with higher dispersal ability than the core population, as observed in different real invasions [41], [42], [44]. Furthermore, the frontal population was less mixed (consisted only of fast dispersers), compared to the population at the core of the invasion where all dispersal abilities were present, as predicted by competition models in which dominant species win and limit weaker individuals' invasion [12], [59]. Finally, spatial sorting was more apparent for more mixed propagules.

The spatial sorting of dispersal abilities was reflected in the mean dispersal rate of the population. At the beginning of the invasion, individuals with better dispersal abilities are low in number as their growth is limited by the individuals with weaker dispersal abilities. However, fast dispersers do not go extinct as they are as competitive as the slow dispersers. As individuals with better dispersal ability reach the front of the invasion, they can grow in number in the open space without any competition against the slow dispersers. The mean dispersal ability at the frontal population therefore increases. This process occurs at every generation during range expansion, and results into an increasing dispersal ability on the expanding edge. The increasing dispersal rate in turn yields an increasing rate of spread, that is, an acceleration of the range expansion. Numerical simulations suggested that the rate of spread between two generations can be approximated using the dispersal ability of the frontal population only. This result is consistent to the findings of Bouin et al. [60]. This result suggests that (1) empirical quantifications of the dispersal ability, such as the mean dispersal rate for all individuals are only accurate for a short period of time and may underestimate the long-term rate of spread of the population (dashed line in Fig. 4) and (2) empirical predictions based on dispersal abilities obtained from the core population can depreciate the real rate of spread.

Unlike other works which took possible mutations of dispersal relevant traits into account [8], [61]–[63] the rate of spread obtained from our model remains bounded as the dispersal abilities are bounded. After the initial acceleration, the population expands at a constant rate. A close formula for the asymptotic rate of spread was derived for the constant asymptotic rate of range expansion (Eq. (14) with Eq. (13)). First, it is worth recalling that the rate of spread was obtained with the assumption that the dispersal ability of the propagule is log-normally distributed. This assumption was used due to different evidences that species-abundance relationships follow a log-normal distribution [53], [64], [65]. The rate of spread, however, can be derived for different propagule distributions simply by using the corresponding cumulative probability function. For instance, for a normally distributed initial propagule, the cumulative probability function is given by 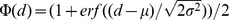 and the rate of spread is given by
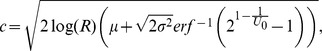
where 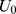 and 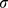 denote the propagule size and compositional diversity, respectively.

Second, the obtained rate of spread is similar to the approximation for the RD model (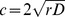). Moreover, the RD result is obtained when a common dispersal rate is shared by all individuals, by letting 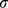 tend to 0. Finally, the expression of the rate of spread suggests that the rate of spread increases with the propagule pressure. This result is in line with the speculation that increasing the propagule size can improve the species spread by providing better suited individuals for invasion [22], [66]. Furthermore, our results are consistent with the findings of Skalski and Gilliam [30] and Yamamura [67] who explored different models to elucidate the importance of different dispersal abilities in a population.

Despite the theoretical progress made here in understanding the acceleration of range expansion in biological invasions, our model only captured one facet of the dynamic nature of dispersal strategies, through the spatial sorting of individuals with diverse dispersal abilities in the initial propagule. Other factors do exists, particularly the evolutionary dynamics of dispersal-relevant traits, which can also affect spreading dynamics. Simulation models have suggested that the eco-evolutionary dynamics of dispersal can either promote [37], [38] or prohibit [36], [38] spatial sorting, and the acceleration of spreading especially in environment with high temporal variability of habitat [36]. Rapid evolution can further affect the ecological dynamics of a population during colonization and spread [68]–[70]. Further models therefore need to take evolutionary detail into consideration to improve the prediction of range dynamics under local and global environmental changes.

To conclude, our results suggest that the variety of dispersal abilities in the initial propagule plays an important role in shaping the range versus time pattern during a population's spread. A biphasic invasion, which consists of two linear range expansions with different rates, resulted from a propagule with two dispersal levels. When the initial propagule was more mixed, the invasion started at a slow rate and then accelerated until a maximal rate of spread was attained. In addition, our results emphasize the importance of census time and locations when estimating the parameters of reaction-dispersal models as data collected from the core of the invasion may underestimate the actual rate of spread.
