# Correction: Spatial Assortment of Mixed Propagules Explains the Acceleration of Range Expansion

**DOI:** 10.1371/journal.pone.0136479

**Published:** 2015-08-19

**Authors:** Andriamihaja Ramanantoanina, Aziz Ouhinou, Cang Hui

In the Results section, there is an error in the fourth equation in the section titled “Propagule with two dispersal abilities” and in the eleventh equation in the section titled “Propagules with multiple dispersal abilities.” In both equations, *γ*_2_ appears incorrectly outside the parentheses. Please view the complete, correct equations here:

$$c\approx \sqrt{2\;\ln(R)d_{2}^{2}}\left(1+\frac{\ln(R)}{12}\gamma_{2}\right),$$ (4)

$$c=\underset{\lambda }{\min}\frac{1}{\lambda }\ln(RM(d_{n}),\lambda )\approx \sqrt{2\ln(R)d_{n}^{2}}\left(1+\frac{\ln(R)}{12}\gamma_{2}\right),$$ (11)
